# Three-dimensional pelvic kinematics in healthy, total hip arthroplasty, and lumbar fusion patients using stereo-radiography

**DOI:** 10.3389/fbioe.2025.1628154

**Published:** 2025-09-04

**Authors:** Kathryn H. Colone, Nicole D. Quinlan, Sam R. Mattei, Kevin B. Shelburne, Paul J. Rullkoetter, Douglas A. Dennis, Chadd W. Clary, Casey A. Myers

**Affiliations:** ^1^ Center for Orthopaedic Biomechanics, University of Denver, Denver, CO, United States; ^2^ Colorado Joint Replacement, AdventHealth Porter, Denver, CO, United States

**Keywords:** spinopelvic mobility, functional pelvic tilt, pelvic obliquity, patient-specific, total hip arthroplasty, biplane radiography, *in vivo*

## Abstract

**Introduction:**

Measuring pelvic tilt and pelvic obliquity during functional positions is important for surgical planning of total hip arthroplasty as these orientations affect optimal acetabular cup position and post-operative hip stability. The objective of this study was to compare pelvic tilt, pelvic obliquity, and pelvic mobility across 3 cohorts of age-matched patients: 1) healthy controls 2) THA patients without spinal fusion and 3) patients with instrumented spinal fusions. We hypothesized that (1) the healthy and THA cohorts would demonstrate similar pelvic mobility across the range of position demand and (2) individuals with spinal fusions would have significantly less pelvic mobility than both the healthy and THA cohorts.

**Methods:**

We compared 10 patients in each of these cohorts using stereo radiography to quantify pelvic tilt and pelvic obliquity across a range of clinically relevant poses of varying demand on pelvic mobility.

**Results:**

Results demonstrated that the overall pelvic mobility of the spinal fusion cohort was consistently similar in magnitude to health controls but biased anteriorly by 6.5% and 33.5% compared to the healthy and total hip cohorts, primarily due to less posterior tilting across the functional positions (Healthy: 39.6° ± 10.2°; Spinal fusion: 39.5° ± 7.3°; Total hip: 37.8° ± 7.6°). Obliquity angles varied substantially between some clinically relevant pose combinations. Low and high coronal plane mobility patients were identified in each of the three cohorts, with mobility ranging between 4.4° and 28.3°, respectively, across positions.

**Discussion:**

Substantial intragroup variability was exhibited within each cohort across the six functional poses, highlighting the patient-specific nature of the spinopelvic relationship regardless of previous surgery at the hip or spine. The larger pelvic tilt angles demonstrated during more demanding poses in seated and standing highlights the importance of imaging patients in poses that capture the full extent of pelvic mobility.

## 1 Introduction

Interactions between the hip and spine define the physiologic orientation of the pelvis during activities of daily living. The functional orientation of the pelvis, particularly in the coronal and sagittal planes, affects acetabular inclination and anteversion, femoral neck clearance during hip flexion, and hip stability (Ike et al., 2018). The magnitude of pelvic functional orientation across activity, as well as pelvic mobility, or the overall range of motion of the pelvis, have been shown to be patient-specific and vary widely across the population (Pierrepont et al., 2017). These relationships must be considered when patients undergo total hip arthroplasty (THA) to ensure optimal component positioning and allow appropriate hip motion without impingement or dislocation.

Pelvic mobility risk factors for hip instability have been described in the literature. Spinal stiffness, especially at the lumbosacral junction, can cause reduced pelvic motion and compensatory motion of the hip joint (Ike et al., 2018; Klemt et al., 2020). THA patients with pre-existing lumbosacral pathology or spinal fusions typically exhibit reduced pelvic mobility with increased rates of postoperative complications compared to individuals without spinal fusion (Buckland et al., 2017a; Esposito et al., 2018; Heckmann et al., 2018; Vigdorchik et al., 2021a). Consequently, patients with high pelvic tilt (PT) angles during functional activities or limited overall pelvic mobility, particularly those with lumbar spine pathology or instrumented fusions, are at greater risk for postoperative impingement and dislocation (Buckland et al., 2019; DelSole et al., 2017; Esposito et al., 2018; Haffer et al., 2022; Ike et al., 2018; Kanawade et al., 2014; Klemt et al., 2020; Langston et al., 2018; McKnight et al., 2019; Snijders et al., 2021). Additionally, patients with large ranges of PT are also potentially at risk (Vigdorchik et al., 2021b). Hypermobile risk factors include patients demonstrating greater than 13° of posterior pelvic rotation from supine to neutral standing and more than 20° of anterior rotation transitioning from standing to flexed seated (Sharma and Vigdorchik, 2021; Vigdorchik et al., 2021a).

Some surgeons perform pre-operative clinical assessments to characterize pelvic mobility by quantifying PT from lateral radiographs captured in static positions, including neutral or extended standing, neutral or flexed seated, and supine position (Buckland et al., 2019; Luthringer and Vigdorchik, 2019; Pierrepont et al., 2017; Vigdorchik et al., 2021b). However, this practice is not uniformly accepted and is commonly reserved for patients with diagnosed spinopelvic deformity. Additionally, identifying specific bony landmarks on lateral radiographs, such as the sacral slope or the anterior superior iliac spines (ASIS) and pubic symphysis needed to determine the pelvic plane can be difficult. The optimal static positions and pathological indicators to assess pelvic mobility remain unclear.

While recent literature has focused on pelvic orientation in the sagittal plane, pelvic obliquity (PO) in the coronal plane also affects acetabular cup positioning during THA, with a significant one-to-one relationship between PO and acetabular inclination (Asai et al., 2021). Variation in PO is common in patients undergoing THA and may be associated with leg length discrepancies (LLD) (Kikuchi et al., 2023; Moharrami et al., 2023; Takemoto et al., 2023; Zhou et al., 2012). PO may also result from spinal deformities such as scoliosis, hip contractures, and muscle weakness, which may not be evident in THA preoperative evaluations. Studies have suggested that patients with greater than 6° PO may require rebalancing of the pelvis to promote post-operative stability (Moharrami et al., 2023; Zhou et al., 2012). Although higher PO angles are commonly seen in patients with hip and spine pathology, best practices for preoperative PO assessment and its surgical implications have yet to be elucidated.

Understanding the differences in PT and PO between patient cohorts during demanding functional positions could provide valuable information to help guide THA component positioning, improve patient outcomes, and limit postoperative adverse events. Biplane radiography is the gold standard to quantify joint kinematics in living patients with high accuracy, with multiple studies reporting submillimeter accuracy in pose quantification (Kapron et al., 2014; Martin et al., 2011; Setliff and Anderst, 2024; Tsai et al., 2013). However, few studies have utilized this technique to investigate the pelvis *in vivo* (Johnson et al., 2022; Tsai et al., 2013). With two imaging planes, biplane radiography provides three-dimensional data which allows for increased accuracy in identifying pelvic kinematics, compared to when using standard two-dimensional radiographs. This methodology allows for a robust and comprehensive assessment of pelvic orientation. The objective of this study was to compare pelvic tilt, pelvic obliquity, and pelvic mobility in the coronal and sagittal planes across 3 age-matched cohorts - healthy controls without hip or spine pathology, those with instrumented spinal fusions without THA, and those with THA without spinal fusion - using stereo radiography across a range of clinically relevant poses of varying demand on pelvic mobility. Based on studies with limited evaluation of patient position that rely on lateral radiographs, we hypothesized that (1) the healthy and THA cohorts would demonstrate similar pelvic mobility across the range of position demand and (2) individuals with spinal fusions would have significantly less pelvic mobility than both the healthy and THA cohorts.

## 2 Materials and methods

Thirty participants were enrolled in one of three cohorts and provided informed consent as part of a larger IRB-approved study (University of Denver IRB, Denver, CO, United States) (Age-matched healthy controls, n = 10; Spinal fusion, n = 10; THA, n = 10). Patient demographics are described in Table 1. There were no significant differences between cohorts for age or BMI (p > 0.05).

**TABLE 1:** Patient demographic information.

Demographics	Cohort		
	Healthy (n = 10)	Spinal Fusion (n = 10)	THA (n = 10)
Age at data collection (years)	59.8 ± 9.9	66.0 ± 8.4	67.0 ± 5.6
Sex (% female)	44	60	50
BMI^a^	25.0 ± 3.9	26.6 ± 3.7	24.9 ± 2.9
Single vs Multi-level Fusions^b^ (% Multi-level)	-	60	-
Unilateral vs Bilateral THA (% Bilateral)	-	-	40

Values are reported as mean ± standard deviation

^a^ Body Mass Index (BMI) calculated as weight (kg) divided by height squared (m2).

^b^ Multi-level fusions included four 3-level, one 5-level, and one 5+ level fusion.

**FIGURE 1:**
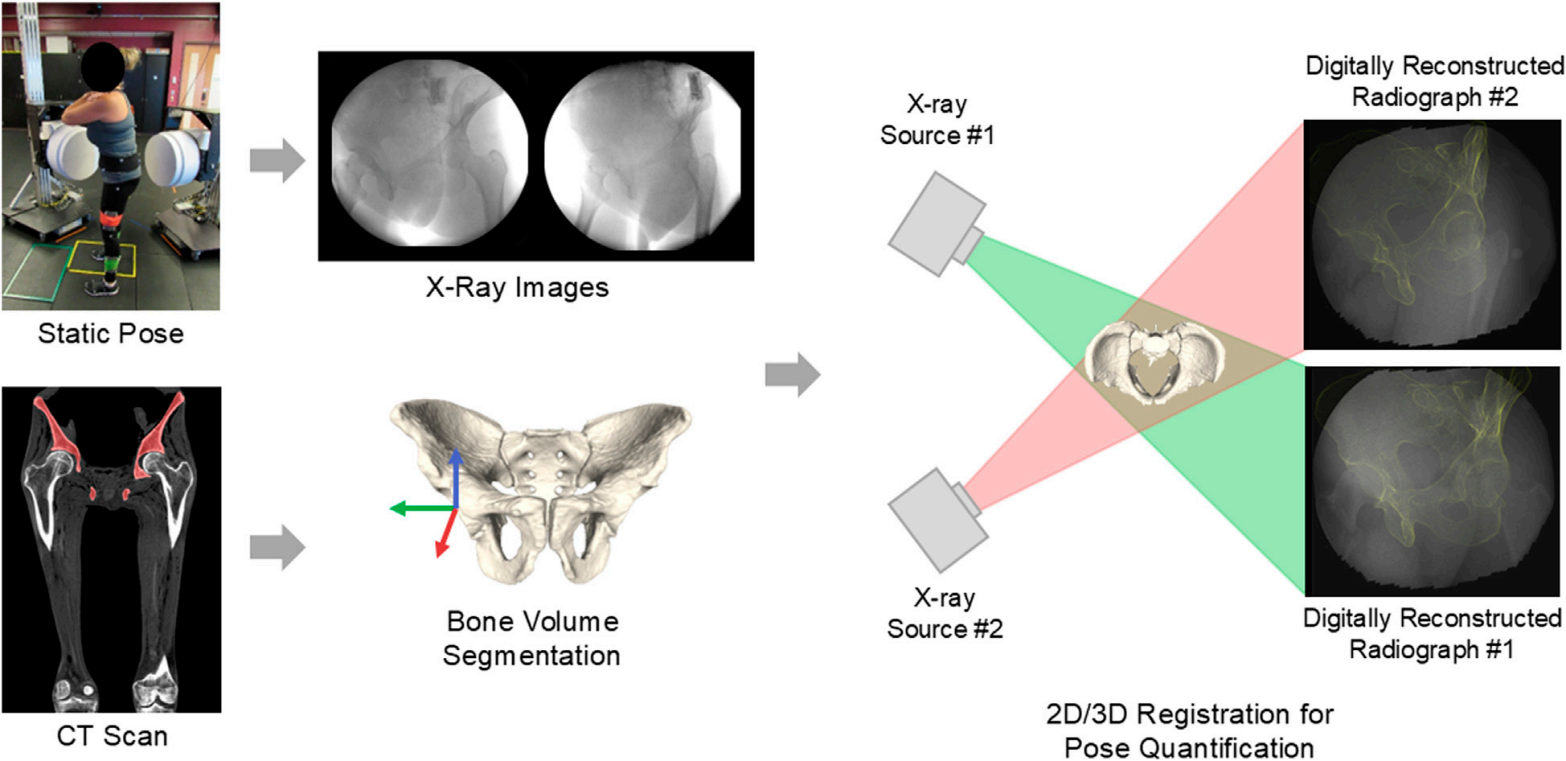
Workflow for biplane radiography and 2D/3D image registration, similar to other biplane radiography studies (Johnson et al., 2022; Martin et al., 2011).

**FIGURE 2:**
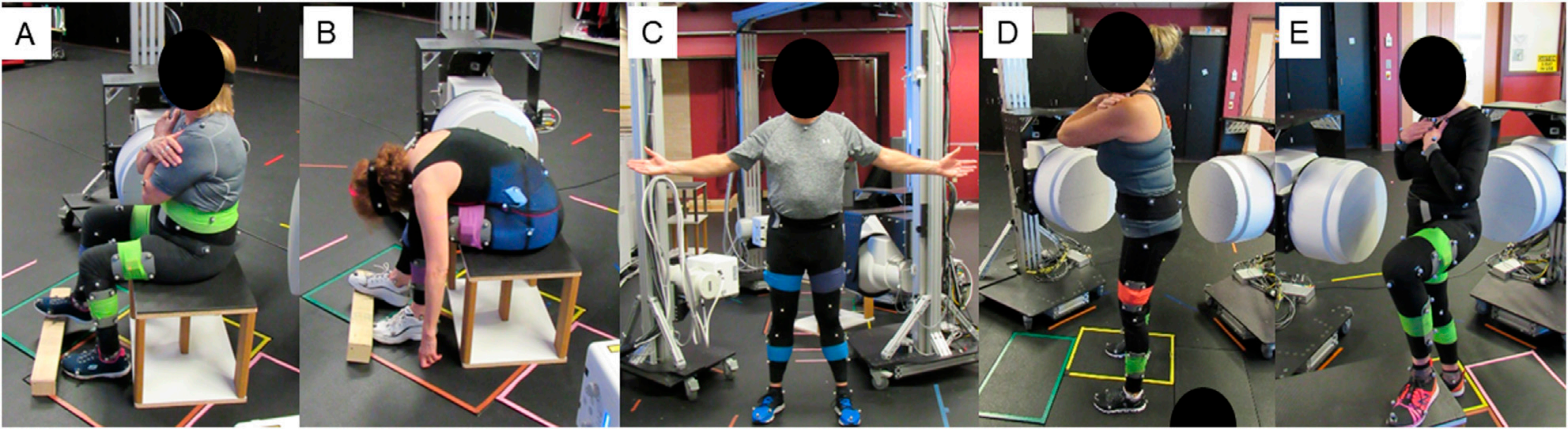
Representative static poses. **(A)** neutral seated **(B)** flexed seated **(C)** neutral standing **(D)** leg extended standing and **(E)** flat back standing.

Data was collected using a high-speed stereo-radiography system (HSSR) with two 40-cm X-ray image intensifiers (TH 9447 QX, Thales, La Défense, FR) integrated with high-definition digital cameras to capture 3D pelvis positions during five static positions (Hamilton et al., 2022; Ivester et al., 2015) (Figure 1). Five static positions were collected that included two seated and three standing poses of varying demand. The two seated positions consisted of neutral and flexed seated. In the neutral seated position, participants were instructed to sit comfortably upright with a non-slouched posture. In the flexed seating position, participants were instructed to fully flex at the waist and reach towards their toes. Patients were seated on a backless bench platform of standard chair height (0.43 m) with feet pointed forwards at shoulder width apart and their right femur elevated by approximately 4° to improve visibility for bone motion tracking (Figures 2A,B).

**FIGURE 3:**
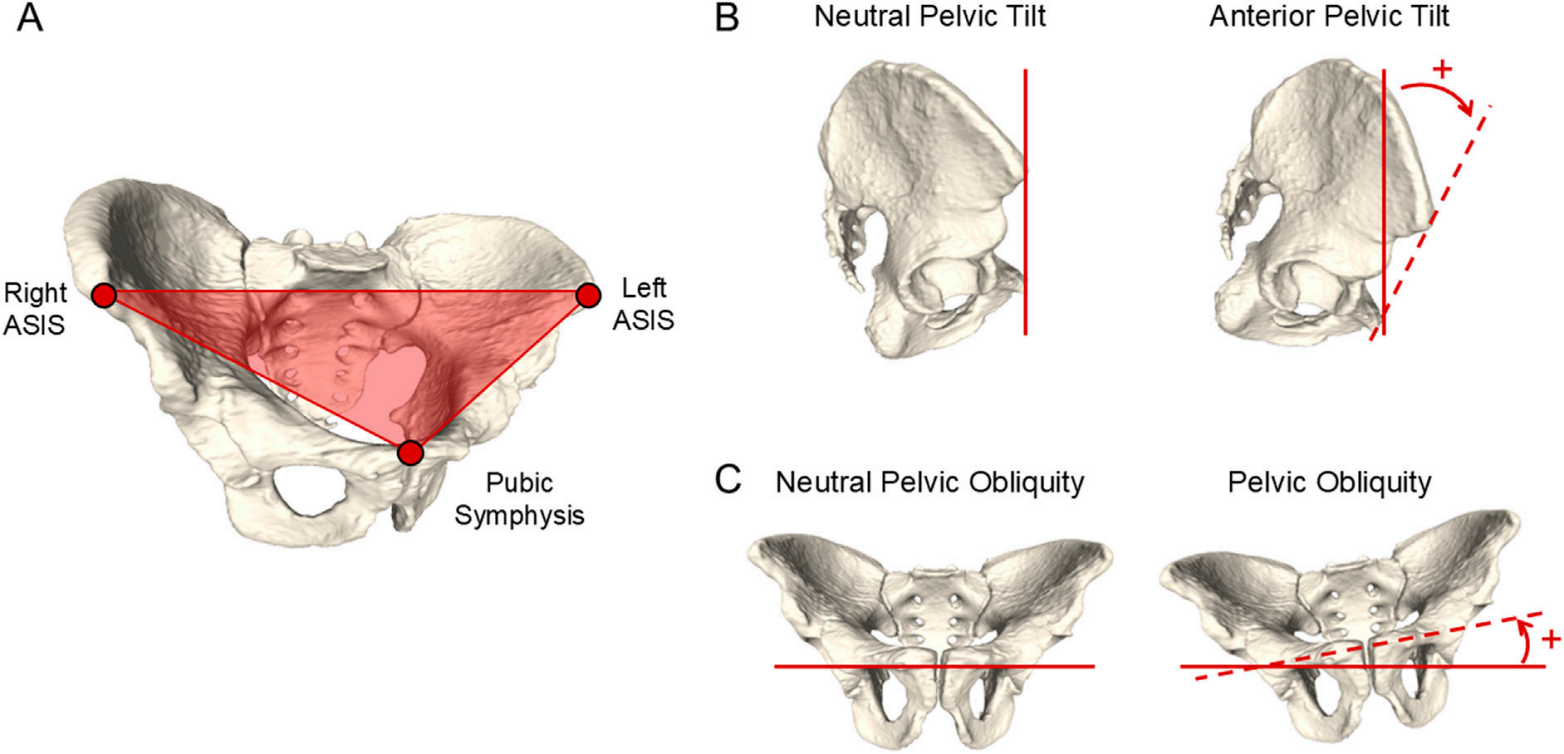
**(A)** Description of the anterior pelvic plane (APP) as defined by the ASIS points and pubic tubercle, used to quantify pelvic tilt. **(B)** Representation of PT angle as seen in the sagittal view, and **(C)** PO angle as seen in the coronal plane.

The standing positions included (1) neutral standing (Figure 2C), (2) flatback standing, where patients were instructed to roll their pelvis posteriorly to achieve as much of a lumbar flat back position as possible (Figure 2D), and (3) hip at 90° with back extended. For this pose, patients raised their non-dominant limb to 90° on a platform and extended backwards at the waist (Figure 2E). For individuals with unilateral THA, their unaffected leg was raised. This position was selected due to its similarity to protocol from preoperative planning technology (Previ et al., 2023). Participants with unilateral THA reported no pain in their non-operative joint and were able to perform all activities pain free.

All patients received CT scans within 1 week of the data collection as part of the study protocol. CT scans were performed from the top of the pelvis to mid knee with varying slice thickness (0.625–5.0 mm). Patient-specific pelvis anatomy was reconstructed from CT images in ScanIP (Simpleware ScanIP, Synopsys Inc., Sunnyvale, CA, United States) and a temporary coordinate system was established for the pelvis by manually landmarking the ASIS points and the pubic tubercles. The superior-inferior axis was oriented parallel to the anterior pelvic plane (APP), defined by the left and right ASIS points and the midpoint of the pubic tubercles (Figure 3). The mediolateral axis connected the left and right ASIS points, directed towards the right. The anteroposterior axis was orthogonal to the superior-inferior and mediolateral axes. The pelvis coordinate system was then re-established algorithmically to remove uncertainty associated with manual landmarking, by identifying the ASIS points as the most anterior point on the left and right pelvis that was superior to the hip center. The pubic tubercles were the most anterior points on the left and right pelvis inferior to the hip center, and the pubic symphysis was calculated as the midpoint of the pubic tubercles.

3D pelvis geometries in their anatomic coordinate systems were used to track bone motion during functional poses using Dynamic Stereo X-ray (DSX) software (HAS-Motion, Kingston, ON, CA) for 2D/3D image registration (Zhou et al., 2012). Pelvic tilt was measured as the angle between the APP and a global vertical reference frame in the sagittal plane, with positive PT indicating anterior tilt. Pelvic obliquity was defined as the angle between the line passing through the hip centers and the global horizontal reference frame in the pelvis’s coronal plane, with a positive PO angle indicating the left hip oriented higher than the right hip (Figure 3) (Karkenny et al., 2021). PT and PO were calculated for each patient in five static positions and the supine position using custom MATLAB code (MathWorks, Natick, MA, United States). For the supine position, PT was defined as the angle between the APP and the horizontal axis, defined by the CT scanner.

Pelvic tilt mobility in the sagittal plane was calculated for each patient as the difference between the most anterior and most posterior PT measured across all positions for each patient. Similarly, coronal plane mobility was calculated as the difference between the most positive PO angle and most negative PO angle across positions for each patient. Pelvic mobility was analyzed for clinically relevant pose combinations including: (1) neutral seated-to-flexed seated, (2) flexed seated-to-neutral standing, (3) neutral seated-to-neutral standing, and (4) supine-to-neutral standing.

Data was processed for a total of 29 individuals, as one participant from the healthy cohort did not complete the CT scan. A two-way analysis of variance (ANOVA) with independent factors of cohort (healthy, spinal fusion, THA) and position (supine, neutral seated, flexed seated, neutral standing, flat back standing, extended standing) was applied to PT and PO. Post-hoc comparisons were applied via Tukey honest-significant-difference tests. Significance was set at a *P* value <0.05.

## 3 Results

Pelvic tilt and pelvic obliquity were quantified during six positions for THA, spinal fusion, and healthy cohorts. Pelvic tilt and coronal plane mobility were defined for each patient based on the range of PT and PO demonstrated by each patient, respectively.

### 3.1 Pelvic tilt

Several statistically significant differences were observed between positions for each cohort (Table 2). All cohorts demonstrated significant differences in PT between the supine-to-neutral seated, neutral seated-to-flexed seated, and flexed seated-to-standing pose combinations (p < 0.05). Additionally, PT between the supine and flat back standing positions were significantly different for the healthy and THA cohorts. The THA subjects also showed significant differences between supine and extended standing, and the spinal fusion cohort indicated significant differences between supine and flexed seated (p < 0.05).

**TABLE 2:** Average (±SD) change in PT for clinically relevant position combinations for all cohorts.

Pose combination	Healthy		Spinal fusion		THA	
	ΔPT	*P-*value^a^	ΔPT	*P-*value	ΔPT	*P-*value
Supine to neutral standing	−9.5 ± 5.4	0.59	−8.4 ± 2.1	0.526	−11.6 ± 8.7	0.294
Supine to neutral seated	−22.7 ± 6.1	<0.01	−18.2 ± 8.4	<0.01	−25.6 ± 8.6	<0.001
Neutral seated to neutral standing	−13.2 ± 8.6	0.238	−9.8 ± 13.1	0.354	−14.0 ± 10.8	0.127
Neutral seated to flexed seated	35.2 ± 7.7	<0.001	36.5 ± 7.6	<0.001	33.1 ± 5.3	<0.001

Values are reported as mean ± standard deviation.

^a^ Probability value for comparison of pelvic tilt between two static poses using a Tukey honest-significant-difference test.

**FIGURE 4:**
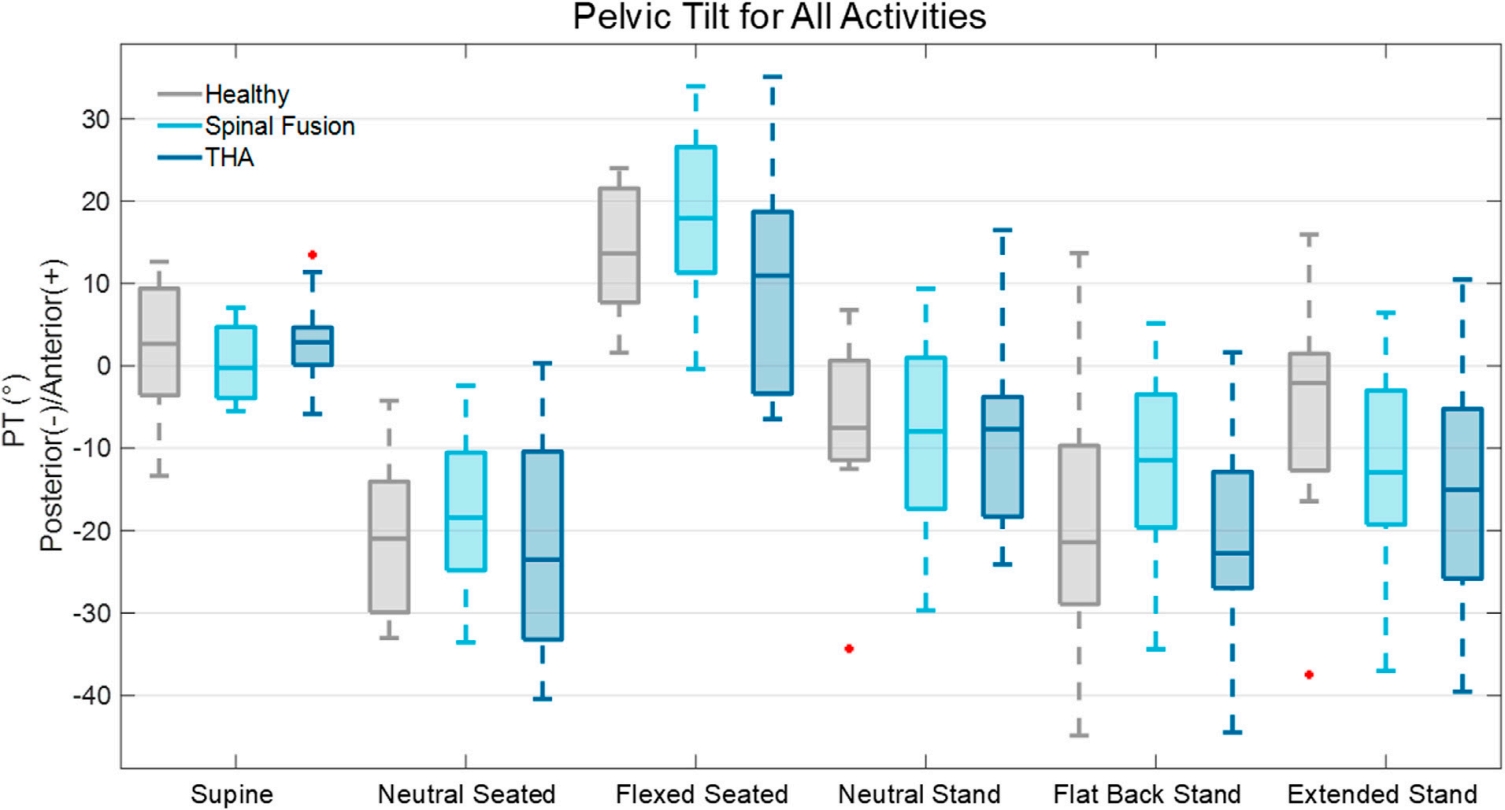
Box plot representation of PT across six static activities for the healthy controls, spinal fusion, and THA cohorts.

All cohorts demonstrated similar PT trends across the six positions, with anterior mean PT for the supine and flexed seated positions, and posterior mean PT for all other positions (Figure 4). The neutral seated position exhibited the most posterior PT out of all positions. The spinal fusion cohort demonstrated the most anterior average PT for the neutral seated and flexed seated poses, and the least posterior tilt compared to the healthy and THA cohorts during the flat back standing pose (Healthy: −18.4° ± 17.3°; Spinal fusion: −12.4° ± 12.5°; THA: −20.9° ± 13.9°). In the supine position, the healthy controls showed the largest range PT range between subjects (Healthy: −13.4° to 12.6°; Spinal fusion: −5.5°–7.0°; THA: −5.9°–13.5°). A high range of PT was observed within all cohorts for the flexed seated activity (Healthy: 1.6°–24.0°; Spinal fusion: −0.39°–33.9°; THA: −6.5°–35.1°).

**FIGURE 5:**
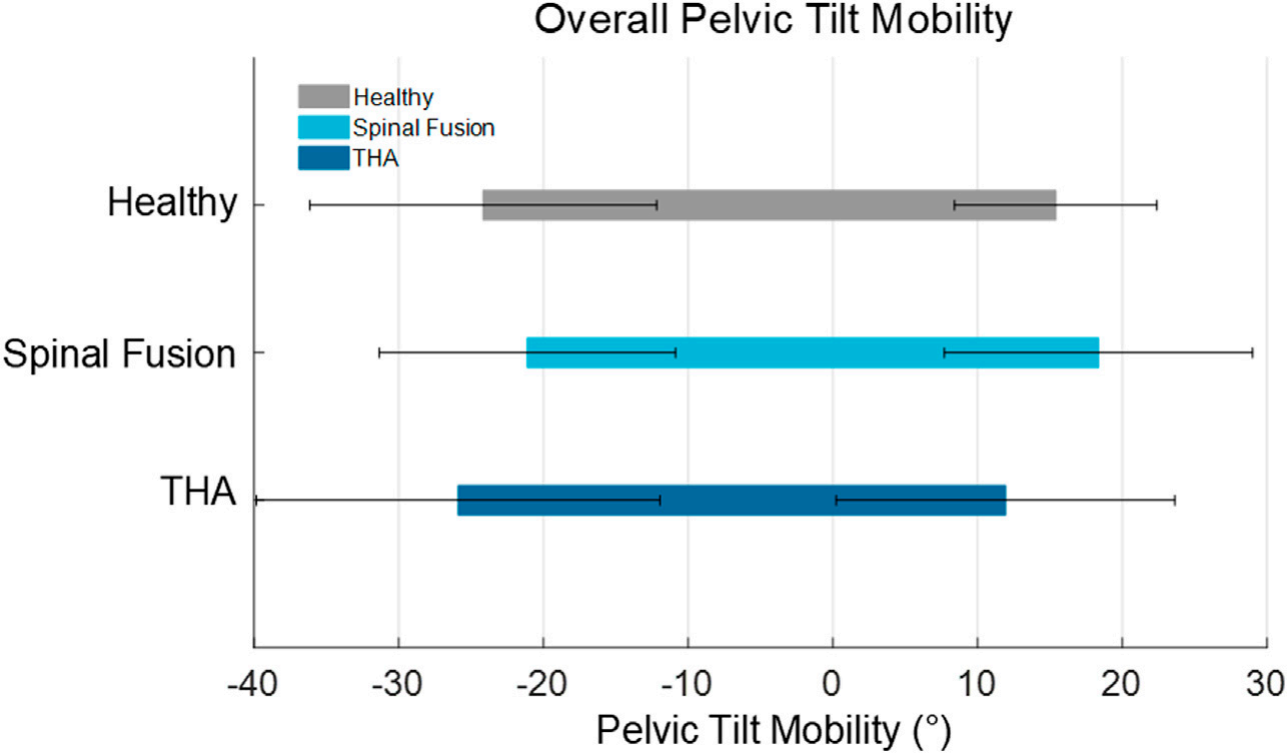
Overall pelvic tilt mobility represented by the average (±SD) most anterior and most posterior PT across all static activities for all patients.

The mean PT mobility across the six positions was similar for all three cohorts (Healthy: 39.6° ± 10.2°; Spinal fusion: 39.5° ± 7.3°; THA: 37.8° ± 7.6°). The average PT mobility of the spinal fusion cohort was biased anteriorly by 6.5% and 33.5% compared to the healthy and THA cohorts, respectively, primarily due to less posterior tilting demonstrated across the functional positions (Figure 5). For clinically relevant pose combinations, similar changes in PT were observed between the neutral and flexed seated positions for all cohorts and patients in each cohort achieved greater than 33° of change in PT transitioning from neutral to flexed seated. The spinal fusion group demonstrated the greatest change in PT between the flexed seated and neutral stand positions compared to the healthy controls and THA group, as well as the least change in PT between the standing poses and supine-to-neutral stand combinations (Figure 4; Table 2). Spinal fusion patients consistently demonstrated lower average change in PT between these three positions (Table 2). For the supine-to-neutral standing activity, spinal fusion patients demonstrated 13.1% and 38.1% less change in PT compared to healthy controls and THA patients, respectively. The discrepancies in change in PT were exacerbated between cohorts for the supine-to-neutral seated and neutral standing-to-neutral seated comparisons.

### 3.2 Pelvic obliquity

**FIGURE 6:**
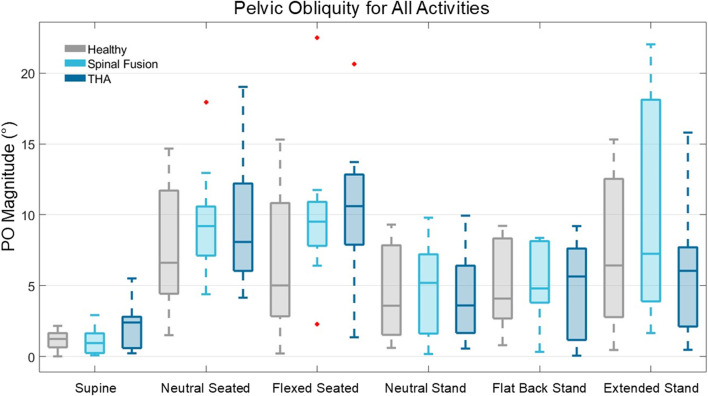
Box plot representation of PO angle magnitude across the six static positions for the healthy controls, spinal fusion, and THA cohorts.

In the supine position, participants demonstrated low variability in pelvic obliquity, with small ranges in PO angle within each cohort (Healthy: −2.2° to 2.1°; Spinal fusion: −2.6°–2.9°; THA: −5.5° to 2.7°) and the average PO angle in this position was close to zero (Healthy: 1.2° ± 0.7°; Spinal fusion: 1.2° ± 1.0°; THA: 2.1° ± 1.6°) (Figure 6). PO variability during other functional positions, however, was higher. In the neutral seated position, participant PO angle varied substantially (Healthy: 1.5°–14.7°; Spinal fusion: 4.4°–18.0°; THA 4.1°–19.0°) and these ranges further increased in the flexed seated position.

Low and high coronal plane mobility patients were identified in all cohorts, with some patients demonstrating as low as 4.4° mobility across positions, while others demonstrated coronal plane mobility as high as 28.3°. For example, a healthy patient demonstrated a change in PO of 24.0° between the neutral and flexed seated poses, indicating the substantial change possible in coronal plane angle.

## 4 Discussion

### 4.1 Pelvic tilt

The main finding of this study was that all cohorts demonstrated similar average overall PT mobility, including spinal fusion patients (Figure 5). This supported our first hypothesis that the healthy controls and those with THA would demonstrate similar pelvic mobility. However, the finding that the spinal fusion patients demonstrated similar pelvic mobility to the other cohorts did not align with our second hypothesis. Previous studies investigating pelvic mobility utilizing lateral radiographs have differed from this finding, reporting reduced pelvic mobility in spinal fusions patients compared to cohorts without spinal fusions (Bernstein et al., 2019; Esposito et al., 2018; Stefl et al., 2017). While we did observe a lack of posterior pelvic tilt in the spinal fusion cohort during functional positions that would help explain the lack of mobility found in previous studies, our results suggest that these patients maintain high levels of overall mobility comparable to cohorts without spinal fusion that is accomplished through an anterior shift in pelvic orientation across positions (Figure 5) due to the presence of the fusion. In our study, the spinal fusion group had on average 12% and 19% more anterior pelvic tilt in the neutral seated position than the healthy and THA cohorts, respectively. The spinal fusion group was also the most anteriorly oriented of the three cohorts in the flexed seated position, with an average of 26% and 72% more anterior tilt than the healthy and THA cohorts, respectively. These results are consistent with a previous study by Behery et al., which reported greater anterior tilt in patients with lumbar fusion and spinal deformity than other cohorts (Behery et al., 2020).

All cohorts demonstrated an average increase in PT from the neutral seated-to-neutral standing pose, which are the most commonly captured positions in preoperative radiographs (Healthy: 13.2° ± 8.6°; Spinal fusion: 9.8° ± 13.1°; THA: 14.0° ± 10.8°). These results agree with findings from previous studies that demonstrated typically the pelvis tilts posteriorly when transitioning from neutral standing to neutral seated to allow for femoral head and neck clearance during hip flexion (Ike et al., 2018). Additionally, based on the Hip-Spine Classification, less than 10° of change in posterior PT from neutral seated to neutral standing is categorized as a stiff spine, which may be an indicator for necessary considerations in acetabular cup placement for inclination and anteversion compared to that for patients with normal mobility (Sharma and Vigdorchik, 2021). On average, the change in PT from neutral seated to neutral standing pose was greater than 10° for the healthy and THA cohorts and less than 10° for the spinal fusion cohort (Table 2). However, at least three patients from each cohort demonstrated this stiff spine behavior of less than 10° change in PT between the two positions.

Several patients in the spinal fusion cohort for this study had multilevel fusions, with 3 of those including sacroiliac (SI) joint fusions (Table 1). Of the 4 patients with single level fusions, 3 of those involved L5-S1, suggesting that while the different types of fusion likely explains some of the variability in the spinal fusion cohort, the spinal fusion patients in this study did not demonstrate the assumed ‘stiff spine’ behavior and reduced mobility that has previously been observed for individuals with these fusions (Buckland et al., 2017b; Malkani et al., 2018). Substantial variability in PT angle and PT mobility was demonstrated for all cohorts across the set of six static poses (Figure 4), suggesting changes in PT do not occur in a predictable manner between patient populations with specific characteristics, such as spinal fusions or THA.

Pelvic orientation in the supine position is of particular interest for guiding acetabular component positioning intraoperatively when a direct anterior approach is utilized. Previous studies have suggested that individuals with spinal fusions or deformities may require special consideration of the functional anterior pelvic plane angle for acetabular cup positioning intraoperatively (Sharma and Vigdorchik, 2021). In this study, pelvic tilt in the supine and neutral standing poses was found to be similar for all cohorts, consistent with findings from previous studies (Babisch et al., 2008). However, several other poses demonstrated PT significantly different from the supine position. All cohorts demonstrated significant differences in PT between the supine and neutral seated poses. There were patients in each cohort who demonstrated greater than 30° change in PT between the supine position and another function pose. More specifically, patients in the healthy and THA cohorts demonstrated 32° and 39° decrease in PT, respectively, from the supine to neutral seated position. These large changes in PT demonstrate the extent to which the pelvis tilts functionally compared to the position in which the acetabular component is positioned during anterior approach THA.

There has been debate on how to assess sagittal mobility for surgical decision-making. Several studies have quantified change in PT solely between the neutral standing and neutral seated positions including the Hip-Spine Classification system recommended to categorize spinal alignment and mobility (Behery et al., 2020; Haffer et al., 2022; Luthringer and Vigdorchik, 2019; Snijders et al., 2021; Vigdorchik et al., 2021a). However, in this study, average changes in PT of over 33° were observed between the neutral and flexed seated positions for all cohorts (p < 0.001) (Table 2). Additionally, the flat back position, selected to encourage a more posteriorly oriented pelvis, resulted in greater average posterior tilt than neutral seated, extended standing, and neutral standing. These results highlight the additional range of motion captured by imaging patients in more demanding positions, representing the full extent of pelvic mobility.

### 4.2 Pelvic obliquity

While no clinically significant metric of PO has been established for surgical correction, previous studies have classified PO greater than 6° in the neutral seated position as severe (Patel and Shapiro, 2015; Zhou et al., 2012). In this study, patients with low and high coronal plane mobility were identified in all cohorts based on changes in obliquity angle between static functional positions. For clinically relevant pose combinations, such as neutral seated-to-flexed seated, 5 of the 10 healthy patients demonstrated a change in PO greater than 6°. When transitioning between the neutral seated and neutral standing poses, the majority of patients in all cohorts experienced a change in PO greater than this 6° threshold.

Relative to the supine position, patients in all cohorts exhibited change in PO ranging 8°–23° during functional poses. Maximum changes in PO from supine to flexed seated within the healthy, spinal fusion, and THA cohorts were 13.8, 22.7, and 23.3°, respectively.

For the THA patients with a unilateral hip prosthesis, the implanted hips were consistently oriented higher than the non-implanted hips with obliquity angles ranging from 0.5° to 6.4° during neutral standing. Studies have shown that a 1° change in PT causes 0.5°–1° change in acetabular cup anteversion, but only a 0.2° change in cup inclination (Asai et al., 2021; Parilla et al., 2019; Yang et al., 2019). However, PO angle is directly proportional to acetabular inclination, with a 1° change in inclination for every 1° change in PO (Asai et al., 2021). These results demonstrated large pelvic obliquity values and patient-specific variability in coronal plane mobility across positions for all cohorts (Figure 6), suggesting the benefit of considering patient-specific PO angle and coronal plane mobility in acetabular cup positioning during THA.

### 4.3 Limitations

There was significant heterogeneity within the THA and spinal fusion cohorts. Spinal fusion cohort participants had varying levels of instrumentation, with some constructs extending to the sacrum and others not including L5-S1 joint fusions. The THA cohort also included individuals with unilateral and bilateral implants. Despite this limitation, several significant differences in pelvic orientation were identified between cohorts with a cohort size of 10 patients. Healthy and THA patients reported no history of low back pathology or hip joint pain, however, additional patient history data was not collected. Lastly, to distinguish both femurs during seated positions, the right femur was elevated using a foot wedge, which may have influenced the pelvic orientation in those positions.

## 5 Conclusion

These results suggest that the presence of an instrumented spinal fusion does not necessarily inhibit pelvic mobility in a predictable manner and that preoperative assessment, particularly with lateral lumbopelvic radiographs in multiple positions, should be considered to guide intraoperative THA acetabular component positioning to improve functional outcomes and limit postoperative complications. High variability in pelvic mobility was identified among each cohort, with each cohort having at least one participant that represented a large deviation from the mean, highlighting the patient-specific nature of the spinopelvic relationship. Future research will investigate how pelvic mobility during dynamic activities of daily living across cohorts compares to ranges established using static radiographs.

## Data Availability

The raw data supporting the conclusions of this article will be made available by the authors, without undue reservation.
